# Knowledge, Attitude, and Practice of Unused and Expired Medication Disposal among Patients Visiting Ayder Comprehensive Specialized Hospital

**DOI:** 10.1155/2020/9538127

**Published:** 2020-08-24

**Authors:** Meles Tekie Gidey, Alem Habtu Birhanu, Afewerki Gebremeskel Tsadik, Abraham Gebrezgabiher Welie, Brhane Teklebrhan Assefa

**Affiliations:** ^1^Pharmacoepidemiology and Social Pharmacy Course and Research Unit, School of Pharmacy, College of Health Sciences, Mekelle University, Mekelle, Ethiopia; ^2^Department of Clinical Pharmacy School of Pharmacy, College of Health Sciences, Mekelle University, Mekelle, Ethiopia; ^3^Department of Pharmacology and Toxicology, School of Pharmacy, College of Health Sciences, Mekelle University, Mekelle, Ethiopia

## Abstract

**Background:**

Accumulation of unused and expired medicines at home is a source of environmental hazards and public health problems due to lack of awareness on appropriate medicine disposal methods. Therefore, the study was conducted to assess the knowledge, attitude, and practice of unused and expired medicine disposal among patients visiting Ayder Comprehensive Specialized Hospital.

**Methods:**

A descriptive cross-sectional study was conducted among 384 patients who visited Ayder Comprehensive Specialized Hospital outpatient pharmacy from April to June 2019. Convenience sampling was used to recruit the participants, and data was collected by a face-to-face interview using a structured questionnaire. The data were entered and analyzed by using SPSS version 21.0. Descriptive statistics on sample characteristics was computed, and results were presented in the form percentage using table and statements.

**Results:**

Out of the 384 respondents included in the study, 205 (53.4%) of them were males. More than half (199 (51.8%)) of the respondents did not correctly knew about medicine waste, and 233 (60.7%) of them did not have any prior information regarding medicine waste disposal instruction. But 351 (91.4%) of the participants correctly responded that inappropriate unused and expired medicine disposal can cause environmental harm. Above half (218 (56.8%)) of the respondents “agreed” about the potential risks related to having unused/expired medicines at home, and 206 (53.6%) of them “strongly agreed” that children are more vulnerable. One hundred fifty-nine respondents had unused/expired medicines in their homes. The most commonly used disposal practice for unused medicines were throwing them in a household trash as reported by 297 (77.3%) followed by flushing unused medications in toilet/sink 152 (39.6%). Throwing them away in household garbage and flushing them in toilet/sink were also the most commonly preferred disposal practice for expired medicines.

**Conclusion:**

The majority of the study participants dispose unused and expired medicine in household garbage and toilet/sink. This is against the recommendations of both national and international policies and guidelines on safe and appropriate pharmaceutical waste disposal.

## 1. Introduction

The use of prescription and over the counter medicine is increasing globally and is expected to reach over 4.5 trillion doses in 2020, a 24% increase from 2015 [1]. However, all the prescribed medicines may not be consumed by the patient, and large quantities of the medications remained unused or expired [2]. There are many reasons for medication accumulation in households such as drug adverse effects, change in dosage/regimen, improvement from illness, expiration of the medicine, promotional influence by manufacturers, physician prescribing practices, dispensing practices, and patient nonadherence [2–5]. Patients may also keep medicines in their house because they need to use it in the future [6, 7]. The accumulation of leftover, unused, or expired medicines at home can be a threat to environmental contamination due to lack of awareness on appropriate medicine disposal methods [8].

Unused and expired medicines can be introduced into the environment when leftover medicines are thrown to household trash through landfill and the liquid medications through the sewerage system. The entry of these medications into the ecosystem can cause diverse environmental hazards. For example, contamination of water bodies by estrogen contraceptives even in trace amount impairs sexual development and feminization of fishes affecting their reproduction [9, 10]. Exposure to diclofenac has been associated with a substantial decline in the population of vultures according to a report from Pakistan [11]. Exposure to expired/degraded tetracycline has been reported to cause renal tubular damage [12], and the presence of antibiotic in water has been associated with antibiotic resistance, and in the long run, it can cause genetic effects in marine life and human being [13]. In addition, inappropriately accumulating leftover household medicines and inappropriate disposal of unused and expired medicine can cause accidental poisoning of young children and has potential for abuse by adolescents specially when the medicines are habit forming [14, 15].

Expired and unused medicines are “unwanted medicines” and need to be disposed safely. According to a study report from Harar city in Ethiopia and other studies on household disposal of unused and expired medicines around the world, the most commonly used disposal methods were pouring down to sewerage system or sink for liquid medicines and throwing them into household garbage's for solid dosage forms [3, 4, 16–21]. This is against the recommendations of the United States Food and Drug Administration and the Ethiopian “medicine waste management and disposal directive” on safe and appropriate pharmaceutical waste disposal [22, 23]. Therefore, proper and safe disposal of expired or unused medicines is highly important to prevent harms to wildlife due to environmental contamination, poisoning, and spread of antimicrobial resistance associated with inappropriate medicine waste disposal.

The Ethiopian food, medicine, and health care administration and control authority (EFMHACA) now Ethiopian Food and Drug Administration (EFDA) issued “medicine waste management and disposal directive” in 2011 with the intention of protecting the public and the environment from health risks and hazards of medicines waste by ensuring safe management and disposal practice [23]. In 2013, the authority issued additional directives on healthcare waste management directive mainly intended for the appropriate management of wastes generated from health facilities, and it includes all types of wastes generated including pharmaceuticals. This directive details about waste minimization, segregation, collection, storage, and treatment. There are also details about which method of waste disposal is appropriate for a given type of healthcare waste [24]. However, both directives' focus area is limited to medicine and other wastes generated from health facilities, as such little concern is given to medicine wastes introduced into the environment from leftover or unused and expired in the households. There are also few awareness creation campaigns regarding safe medicine waste disposal methods and practices. Therefore, it is imperative to know the patients' knowledge, attitude, and practices of appropriate disposal of unused and expired household/leftover medicines as an input for policy makers in this area.

Therefore, the aim of this study is to assess knowledge, attitude, and practice of unused or expired household medicine disposal among patients attending outpatient pharmacy in a comprehensive Specialized Teaching Hospital in Northern Ethiopia.

## 2. Method

### 2.1. Study Setting and Period

The study was conducted from April to June 2019 among patients attending outpatient pharmacy in Ayder Comprehensive Specialized Hospital (ACSH). ACSH is found in Mekelle city, 783 km North from Addis Ababa, the capital city of Ethiopia. The hospital serves about 8 million patients from Tigray, Northern Amhara, and Afar regional states, and it is a teaching hospital of Mekelle University. There are six pharmacy units in the hospital: outpatient pharmacy, inpatient pharmacy, Antiretroviral (ART) pharmacy, emergency pharmacy, operation pharmacy, and delivery pharmacy units.

### 2.2. Study Design, Population, and Sampling

A descriptive cross-sectional study design was employed to conduct the study. All patients above 18 years old and attending the outpatient pharmacy dispensary unit and willing to participate in the study were included. Patients with cognitive and communication problems were excluded from the study. Single population proportion formula was used to determine the sample size of 384 participants, and a convenience sampling method was employed to recruit them. The participants were selected based on their availability in the outpatient pharmacy dispensary to refill their prescriptions. The data collector approaches the patients leaving the dispensary after explaining the purpose of the study and getting their consents. If the approached participant declined to participate in the study, the data collector approaches the next patient consecutively.

### 2.3. Data Collection

The data were collected by a face-to-face interview using a structured interview questionnaire. The questionnaire was developed by reviewing different literatures [4, 16, 19]. Then, it was translated into local language (Tigrigna) and back translated for consistency, and a pretest was done before actual data collection commenced. The data were collected by a fifth-year pharmacy student after getting training regarding the data collection procedure, and the data collection process was supervised on daily basis.

### 2.4. Data Analysis

The collected data were checked and cleaned for completeness then entered and analyzed by using SPSS Version 21.0. Descriptive statistics on sample characteristics and variable of interest was computed and presented in the form percentage using table and statements.

### 2.5. Ethical Consideration

Ethical approval was obtained from the ethics review committee of School of Pharmacy, College of Health Science, Mekelle University, and letter of support was granted from the ACSH administration to conduct the study. Written informed consent was also obtained from each respondent.

## 3. Results

### 3.1. Sociodemographic Characteristics of the Respondents

All the approached 384 respondents agreed to participate in the study. Out of the 384 respondents, 205 (53.4%) of them were males and 180 (46.9%) were single. More than two-thirds (272 (70.5%)) of the study participants were from urban areas, and almost half of the study participants (191 (49.7%)) had college and above academic qualifications (Table 1).

### 3.2. Knowledge about Unused and Expired Medicine Disposal

More than half (199 (51.8%)) of the respondents did not know about medicines waste, and 233 (60.7%) of them did not have any prior information about medicine disposal instructions. But 351 (91.4%) of the respondents correctly responded that inappropriate unused/expired medicine disposal can cause harm. Accordingly, 291 (75.8%), 244 (63.5%), and 349 (90.9%) of the participants explained that inappropriate disposal of medicines can contaminate the environment, kill wildlife, and cause accidental swallow by children, respectively. According to 274 (71.4%) of respondents' suggestions, providing proper guidance on how to dispose unused or expired medicines to the consumer could reduce or minimize the hazardous effect of unused/expired medication. Mass media, physicians, pharmacists, and the pharmaceutical industries are responsible to create awareness to the public about proper disposal of unused and expired medicines as reported by 191 (49.7%), 259 (67.4%), 233 (60.7%), and 184 (47.9%) of the respondents, respectively (Table 2).

### 3.3. Respondents' Attitude towards Unused and Expired Medicine Disposal

Above half (218 (56.8%)) of the respondents “agreed” about the potential risks related with having unused/expired medicines at home, and 206 (53.6%) of them “strongly agreed” that children are more vulnerable to the risks associated with having unused and expired medicines in household. Nearly half, 189 (49.2%) and 180 (46.9%), of the respondents “strongly agreed” and “agreed,” respectively, on the need to initiate an outreach and awareness creation programs on how to dispose unused and expired medicines safely (Table 3). Nearly one-fifth (74 (19.3%)) of the respondents “strongly agreed,” and 177 (46.1%) of them “agreed” that take-back programs of unused and expired medicines should be mandatory (Table 3).

### 3.4. Disposal Practice of Unused and Expired Medicine

One hundred fifty-nine (41.4%) of the respondents were having unused or expired medicines in their households. Improvement in medical condition (99 (62.3%)) followed by change of medication by a prescriber (40 (25.2%)) was the main reason for having unused and expired medicine at home. The most commonly used disposal practice for unused medicines was throwing them in a household trash as reported by 297 (77.3%) followed by flushing unused medications in toilet/sink, 152 (39.6%). Similarly, expired medicines were also disposed by throwing away in household garbage and flushed in toilet as reported by 271 (70.6%) and 155 (40.4%) the respondents, respectively. Near two-thirds (62%) of the respondents reported that they separate unused and expired medicines before disposal, and 313 (81.5%) of the participants dispose unused and expired medicines as it is in its original package (Table 4).

## 4. Discussion

With the increasing production and consumption of pharmaceuticals, accumulation and inappropriate disposal of unused and expired household medicines can cause environmental and public health problems [25]. This can be minimized by implementing medicine disposal policy and guidelines [26–29] and improving awareness of the public on appropriate medicine disposal methods and practices [30]. Therefore, the purpose of this study was to assess the knowledge, attitude, and practice of unused and expired household medicine disposal among patients visiting ACSH outpatient pharmacy.

In this study, 351 (91.4%) of the participants correctly understood the negative impacts of inappropriately disposing unused and expired medicine to the environment. According to their understanding, it can cause environmental contamination, can kill wildlife, and can cause accidental swallow by young children. This finding is higher compared to a prior study conducted in Ethiopia [16] and other studies conducted elsewhere in the world [2, 6, 8, 17, 21, 31]. But it is relatively lower compared to a study conducted in Kabul, Afghanistan [4]. The difference might be related to differences in awareness creation campaign across governments about the negative impact of inappropriate disposal of unused and expired medicines or differences in the educational level of the respondents across the studies.

In the present study, higher proportions of the respondents were having little understanding and information regarding appropriate unused and expired medicine disposal instructions. Only 151 (39.3%) of the respondents heard and were informed about unused or expired medicine disposal instructions. This is relatively lower compared to a study conducted in Ethiopia and elsewhere [2, 14, 16], but higher compared to other studies from Pakistan, India, and Saudi [8, 17, 20]. The difference might be attributed to differences in awareness creation campaigns and programs on safe medicine waste disposal.

Three-fourths of the study participants suggested providing proper guidance on how to dispose unused and expired medicine in order to minimize the impacts of unused and expired medicines. Furthermore, one-third of the participants suggested lowering the quantity of medicines prescribed in order to prevent the impact of unused and expired medicines. The finding is consistent with a previous study in Ethiopia [16]. This indicates that health care professionals are less involved in guiding and consulting their patients on how to dispose unused and expired medicines safely.

In this study, more than three-fourths of the participants agreed that there was a lack of adequate information on safe disposal practice of unused and expired medicines. Almost all the participants agreed on the need to have an awareness creation program on how to dispose unused and expired medicines properly. They suggested that mass media, the pharmaceutical industries, physicians, and pharmacists are responsible in creating awareness in the public about the proper disposal of unused and expired medicines. The finding is in consistence with a study done in Harar city [16] and other studies conducted elsewhere [2, 8, 14, 20, 21]. According to the studies, health care professionals were not informing their patients regarding proper disposal of unused and expired medicines, and the majority of the participants were demanding further awareness creation programs. This all indicates the need to implement an awareness creation campaign through appropriate medium to promote proper medicine disposal practices.

In the present study, nearly two-thirds of the respondents agreed on the need to have mandatory medicine take-back program. This is in consistence with previous studies conducted in Ethiopia and elsewhere [3, 7, 14, 16]. Despite higher inclination towards medicine tack-back program in the present study, there is no established medicine take-back system in Ethiopia. This positive attitude towards medicine take-back program might be a good opportunity to minimize the negative impact of inappropriate medicine disposal if the government takes the initiative for the establishment of the system.

In this study, 159 (41.4%) of the respondents had unused or expired medicines in their household. This is higher compared to studies conducted in Ethiopia (29%) [32] and Pakistan (35.3%) [8]. But many other studies reported a higher level of unused and expired medicine storage practice in their households [2, 4, 6, 7, 14, 16, 17, 31, 33, 34]. The difference might be related to differences in medicine or information access across the studies. The high level of unused and expired household medicine storage practice can be a source of accidental poisoning by young children and sources of inappropriate drug use and self-medication practice.

Improvement in medical condition, change of medicines by prescriber, keeping for future use, and intolerable medicine side effect were the most commonly cited reasons for having leftover medicines. This was in agreement with other studies where improvement in medical condition, keeping for future use, regimen change, and drug side effect are among the reasons for keeping unused medicines in household [2, 17].

In the present study, throwing away the medicines in household garbage and flushing in toilet/sink were the most commonly preferred disposal practices for both unused and expired medicines. The finding is in agreement with studies conducted in Ethiopia [16, 34] and elsewhere in the world [4, 6, 20, 21, 31, 35]. In our study, burning leftover medicines were the other means of disposing for both unused and expired household medicines as reported by nearly two-thirds of the respondents. But very few participants were reported returning unused and expired medicines to health care facilities/healthcare providers. This is in agreement with a previous study from Ethiopia [16]. The reason might be due to a lack of awareness on how to appropriately dispose unused/expired medicines. But other studies from European countries and elsewhere reported higher level of unused and expired medicine that had been returned to the pharmacies or healthcare facilities [30, 33, 36, 37]. The low level of unused and expired medicine returning practice in our study might be related to a lack of established medication take-back program. Overall, the mentioned unused and expired medicine disposal practices in the present study were not as recommended by the Ethiopian “medicine waste management and disposal directive” [23].

More than eighty percent of the participants discarded or were willing to discard unused and expired medicines in their original packing. This finding was in consistence with previous studies in Ethiopia [16, 34] and elsewhere [19, 31]. This practice was not in line with the recommended ways of discarding expired and unused medicines and can be a source of accidental poisoning and abuse if the disposed medicines are found to be habit forming [23].

The unused/expired pharmaceutical waste disposal practice is against the recommendations of the national pharmaceutical waste disposal directives. We believe implementing medication tack-back scheme and creating public awareness to return any unused/expired medicines to healthcare facilities or designated collection site are important to prevent inappropriate pharmaceutical disposal to the environment.

### 4.1. Limitation of the Study

This is a descriptive cross-sectional study; as such, it is difficult to identify factors associated with the participants' knowledge, attitude, and practice of unused and expired medicine disposal practices. Since the study was conducted among patients visiting a hospital, there might be discrepancies with practices within the community.

## 5. Conclusion

The majority of the study participants dispose unused and expired medicine in household garbage and toilet/sink. This is against the recommendations of both national and international policies and guidelines on safe and appropriate pharmaceutical waste disposal. This can be sources of environmental hazards and public health problems. Therefore, implementing an awareness creation campaign through an appropriate medium of communication on the appropriate medicine waste disposal method and the impact of inappropriate disposal practice is crucial to protect the public and the ecosystem.

## Figures and Tables

**Table 1:** Sociodemographic characteristics of participants in Ayder Comprehensive Specialized Hospital, Mekelle, Ethiopia, July 2019 (*n* = 384).

Variables	Frequency (%)
Gender	
Male	205 (53.4)
Female	179 (46.6)
Age (years)	
18-24	95 (24.7)
25-35	157 (40.9)
36 and above	132 (34.4)
Residence	
Rural	112 (29.2)
Urban	272 (70.8)
Marital status	
Single	180 (46.9)
Married	175 (45.6)
Divorced	15 (3.9)
Widowed	14 (3.6)
Educational status	
No formal education (illiterate)	52 (13.5)
Elementary school (1-8)	46 (12.1)
Secondary (9-12)	95 (24.7)
College and above	191 (49.7)
Occupations	
Self-employed	142 (37.0)
Governmental employee	92 (24.0)
Student	88 (22.9)
Housewife	62 (16.1)

**Table 2:** Respondents' knowledge of unused and expired medicine disposal in Ayder Comprehensive Specialized Hospital, Mekelle, Ethiopia, July 2019 (*n* = 384).

Questions	Frequency (%)
Have you ever heard about medicine waste?	
Yes	185 (48.2)
No	199 (51.8)
If yes, which one of the following can be considered as medicine wastes? (*n* = 185)^∗^	
Expired medicines	184 (44.9)
Leftover medicines due to some reasons	150 (39.1)
Damaged medicines that cannot be used	165 (43.0)
Once opened medication and beyond their recommended use date	176 (45.8)
Do you have to check the expiry date of your medication?	
Yes	244 (63.5)
No	140 (36.5)
Have you ever read or heard medicine disposal instructions?	
Yes	151 (39.3)
No	233 (60.7)
Do you think that inappropriate unused medicine disposal can cause harm?	
Yes	351 (91.4)
No	33 (8.6)
What do you think is the possible harm associated with inappropriate medicine disposal?^∗^	
It can contaminate the environment	291 (75.8)
It can kill wildlife	244 (63.5)
Can cause accidental swallow by children	349 (90.9)
How could the hazardous effect of unused and expired medicines be minimized or controlled?^∗^	
Providing proper guidance to the consumer	274 (71.4)
Prescribing in quantities and for duration that ensure patient compliance	113 (29.4)
Lowering the quantities of prescribed medicine at a time	128 (33.3)
Donating or sharing the unused medicines	34 (8.9)
Who do you think is responsible to create public awareness about proper disposal of unused and expired medicines?^∗^	
Mass media	191 (49.7)
Physician	259 (67.4)
Pharmacy	233 (60.7)
The pharmaceutical industry	184 (47.9)

^∗^Multiple response percent might exceed 100.

**Table 3:** Respondents' attitude towards unused and expired medicine disposal in Ayder Comprehensive Specialized Hospital, Mekelle, Ethiopia, July 2019 (*n* = 384).

Statements	Strongly agree, *f* (%)	Agree, *f* (%)	Neutral, *f* (%)	Disagree, *f* (%)	Strongly disagree, *f* (%)
Unused and expired medicines present potential risks at home	131 (34.1)	218 (56.8)	14 (3.6)	21 (5.5)	0 (0)
There is lack of adequate information on safe disposal of unused medicine	105 (27.3)	190 (49.5)	39 (10.2)	45 (11.7)	5 (1.3)
Children are more vulnerable to the risks of associated with unused and expired household medicines	206 (53.6)	152 (39.6)	19 (4.9)	7 (1.8)	0 (0)
Doctors and healthcare professionals should provide advice on safe disposal of unused and expired household medicines	49 (12.8)	111 (28.9)	63 (16.4)	114 (29.7)	47 (12.2)
Take-back programs of unused and expired medicines should be mandatory	74 (19.3)	177 (46.1)	41 (10.7)	80 (20.8)	12 (3.1)
Outreach and awareness programs about how to dispose unused or expired medicines should be initiated	189 (49.2)	180 (46.9)	5 (1.3)	10 (2.6)	0 (0)

^∗^ *f* indicates the frequency of responses.

**Table 4:** Respondents unused and expired medicine disposal practice in Ayder Comprehensive Specialized Hospital, Mekelle, Ethiopia, July 2019 (*n* = 384).

Questions	Frequency (%)
Do you or anyone in your household has medications that are no longer needed?	
Yes	159 (41.4)
No	225 (58.6)
If yes, what is the reason for having unused medication? (*n* = 159)^∗^	
Improvement in medical condition	99 (62.3%)
Change of medication by prescriber	40 (25.2%)
Intolerable side effect	17 (10.7)
Keeping for future use	68 (42.8)
What do you do with the unused medicines?^∗^	
Throw away in household garbage	297 (77.3)
Flush unused medications in toilet/sink	152 (39.6)
Keep at home until expired	43 (11.2)
Burn	122 (31.8)
Give to friends or relatives	11 (2.9)
Return back to pharmacy	10 (2.6)
I do not know what to do	16 (4.2)
What do you do with the expired medicines?^∗^	
Throw away in household garbage	271 (70.6)
Flush unused medications in toilet/sink	155 (40.4)
Keep at home	14 (3.6)
Burn	126 (32.8)
Give to friends or relatives	10 (2.6)
Return back to pharmacy	7 (1.8)
I do not know what to do	35 (9.1)
Do you separate unused medicines before disposal?	
Yes	238 (62.0)
No	146 (38.0)
How do you discard?/way of discarding unused and expired medicines^∗^	
Crashed before discarding	55 (14.3)
Diluted with water	87 (22.7)
As it is	313 (81.5)
I do not know what to do	45 (11.7)

^∗^Multiple response percent might exceed 100.

## Data Availability

All datasets from which we derived our conclusion are deposited in SPSS software, and it can be accessed from the corresponding author on reasonable request.
